# Lesions of the Basal Forebrain Cholinergic System in Mice Disrupt Idiothetic Navigation

**DOI:** 10.1371/journal.pone.0053472

**Published:** 2013-01-08

**Authors:** Adam S. Hamlin, Francois Windels, Zoran Boskovic, Pankaj Sah, Elizabeth J. Coulson

**Affiliations:** 1 The Queensland Brain Institute, The University of Queensland, Brisbane, Queensland, Australia; 2 School of Biomedical Sciences, Charles Sturt University, Wagga Wagga, New South Wales, Australia; Nathan Kline Institute and New York University School of Medicine, United States of America

## Abstract

Loss of integrity of the basal forebrain cholinergic neurons is a consistent feature of Alzheimer’s disease, and measurement of basal forebrain degeneration by magnetic resonance imaging is emerging as a sensitive diagnostic marker for prodromal disease. It is also known that Alzheimer’s disease patients perform poorly on both real space and computerized cued (allothetic) or uncued (idiothetic) recall navigation tasks. Although the hippocampus is required for allothetic navigation, lesions of this region only mildly affect idiothetic navigation. Here we tested the hypothesis that the cholinergic medial septo-hippocampal circuit is important for idiothetic navigation. Basal forebrain cholinergic neurons were selectively lesioned in mice using the toxin saporin conjugated to a basal forebrain cholinergic neuronal marker, the p75 neurotrophin receptor. Control animals were able to learn and remember spatial information when tested on a modified version of the passive place avoidance test where all extramaze cues were removed, and animals had to rely on idiothetic signals. However, the exploratory behaviour of mice with cholinergic basal forebrain lesions was highly disorganized during this test. By contrast, the lesioned animals performed no differently from controls in tasks involving contextual fear conditioning and spatial working memory (Y maze), and displayed no deficits in potentially confounding behaviours such as motor performance, anxiety, or disturbed sleep/wake cycles. These data suggest that the basal forebrain cholinergic system plays a specific role in idiothetic navigation, a modality that is impaired early in Alzheimer’s disease.

## Introduction

Alzheimer’s disease results in cognitive impairment, including deficits in memory and spatial navigation [1], [2]. Spatial navigation can be conceptualized as world-centred (allothetic) navigation, which uses information about distal cues independent of the subject’s position, and body-centred (idiothetic/egocentric) navigation, which uses self-movement kinaesthetic information [3], [4]. Patients with Alzheimer’s disease or the prodromal stage of the condition, amnesic mild cognitive impairment (MCI), have been shown to perform poorly on both real space and computerized cued (allothetic) or uncued (idiothetic) navigation tasks [5]–[8]. Furthermore, such tests detect impairment prior to more widely used verbal memory tests [8]. Although the allothetic and idiothetic navigation systems both require the hippocampal formation, it is probable that different neural circuits are involved. For example, lesions to the hippocampal dentate gyrus in rodents or the right temporal lobe in humans cause deficits in allothetic navigation, with lesser impact on idiothetic navigation tasks [9], [10]. Although the nucleus responsible for regulating this latter function has not been determined, various lines of evidence suggest the involvement of the cholinergic neurons of the basal forebrain.

Atrophy of the basal forebrain has been consistently correlated with generalized cognitive decline in Alzheimer’s disease [11]–[13]. Moreover, magnetic resonance imaging has shown that basal forebrain atrophy can be detected in subjects with MCI as early as 4.5 years before the appearance of overt clinical symptoms [14], consistent with the stage at which spatial navigation impairment can be detected in human analogues of the Morris water maze task [5], [6]. Furthermore, non-specific lesions to the basal forebrain and the septo-hippocampal projections via the fornix have been shown to disrupt idiothetic navigation in rodents [15], [16], although the specific cellular network that regulates this response remains to be identified. The basal forebrain comprises a heterogeneous collection of nuclei located in the medial septum, the vertical and horizontal diagonal bands of Broca and the basal nucleus (Meynerts), providing the major cholinergic, GABAergic, and glutamatergic innervation to the hippocampus, as well as subcortical and cortical structures [17]. Of these cell groups, the cholinergic neurons are strongly implicated in learning and memory, particularly attentional processing and the encoding of new memories [18]–[20].

Loss of integrity of the basal forebrain cholinergic neurons is a consistent hallmark of Alzheimer’s disease at autopsy [21]–[25], suggesting that the changes in navigation apparent in MCI and Alzheimer’s disease patients result from the specific loss of cholinergic projection neurons in the basal forebrain. In this study we tested whether loss of these neurons explains deficits in idiothetic navigation. A saporin toxin conjugated to an antibody recognizing the p75 neurotrophin receptor (p75^NTR^) was used to create a specific lesion of the cholinergic neurons of the basal forebrain in mice, and their uncued spatial navigational abilities were quantified. We found that mice with a basal forebrain lesion displayed very disorganized behaviour in a passive avoidance navigation task lacking extramaze cues, but performed no differently from control animals on a range of other tests including contextual conditioning and working spatial memory. These results demonstrate that impaired idiothetic navigation is directly attributable to the loss of cholinergic neurons of the basal forebrain.

## Methods

### Animals

Experimentally naive male C57Bl6 mice (8–12 weeks of age) were housed in groups of four in plastic cages. Mice were maintained on a 12 hour light/dark cycle (lights on at 7∶00 a.m.), with food and water provided ad libitum. All procedures were approved by the University of Queensland Animal Ethics Committee and conducted in accordance with the Australian code of Practice for the Care and Use of Animals for Scientific purposes.

### Surgery

8 week old male C57Bl/6J mice (22–24 g) were anaesthetized by intraperitoneal (i.p.) injection of ketamine (100 mg/kg) and the muscle relaxant xylazine (10 mg/kg). Each mouse was then placed in a stereotaxic frame (David Kopf Instruments), with the incisor bar maintained at −3.3 mm below horizontal to achieve a flat skull position. Bilateral infusions of murine-p75^NTR^-saporin (mu-p75-SAP; 0.4 µg/µl; Advanced Targeting Systems) or control rabbit-IgG-saporin (Rb-IgG-SAP; 0.4 µg/µl) were performed using a 30G needle attached to a 5 µl Hamilton syringe. The needle was lowered into the lateral ventricle (A-P, −0.3 mm; M-L, ±1.0 mm; D-V, −2.0 mm from Bregma) [26], and infusions were conducted over 5 minutes (0.2 µg/ventricle); the needle was then left in place for 10 minutes to allow for diffusion. Immediately after surgery, mice were injected subcutaneously with the analgesic torbugesic (2 mg/kg), and the antibiotic Baytril (5 mg/kg).

### Behavioural Testing

Fourteen to twenty-one days after surgery mice were subjected to a range of behavioural tests. Separate cohorts of mice were used to analyse performance in the place avoidance and contextual fear conditioning tasks. Prior to these behavioural tests mice were tested for levels of anxiety using the light/dark test, video tracking of sleep/wake cycles, or the Y-maze. Following contextual fear conditioning, tests of motor integrity were performed using a Rota-rod.

#### Place avoidance

The place avoidance task was a modification of test first described by Cimadevilla et al. [27] in which all intramaze and extramaze cues were removed to minimize allothetic navigation (spatial relationships to external cues) and to enhance the use of idiothetic navigation (self-motion kinaesthetic cues). The apparatus (Bio-Signal Group) consisted of a grey opaque arena (80 cm diameter, 20 cm high) placed on a square grid of parallel metal rods (4 mm diameter, 5 mm apart) on a stainless steel table 1 metre above the floor. The entire apparatus was placed inside a 2 metre×2 metre enclosure consisting of plain grey sound-attenuating curtains. Light levels were maintained at 35 Lux throughout the experiment. During all procedures a 60 dB white noise was used and 1% acetic acid solution was wiped over the apparatus before the mice entered the arena. An overhead camera (Flea2, Pointgrey) was used for tracking the position of the animal in the apparatus. A shock could be triggered when the animal entered a specific sector of the arena (Tracker software, Bio-Signal Group).

On day one all mice underwent one exploration session lasting 5 minutes in which they could freely explore the arena without any shocks being applied. For each phase of the experiment the mice entered the arena directly opposite the shock zone, and were immediately returned to their home cage following trials. After 1 hour, mice were given a single 5 minute training session in which they learned to avoid a 60° stable shock zone. During training the mice received a 0.4 mV foot-shock for half a second when they entered the 60° shock zone, with an entrance delay of 500 ms, and an inter-shock interval of 1 second. To determine place memory for the shock zone, mice were allowed to freely explore the arena 24 hours after the training session without any shocks being applied. During testing, behaviour was measured by the number of entrances into the shock zone over a 5 minute period.

The positional data from the testing session were used to measure avoidance of the shock sector. This analysis was based on the fraction of time each animal spent opposite the shock sector and the spread of the area explored. We first measured the angle between the shock area and the preferred position of the animal and the range of the area in which the animal spent most of its time. Subtracting these two angles provided a measure of the constraint of the animal’s position away from the shock area. This allowed us to differentiate the time spent away from the shock area from random navigation.

#### Y-maze

The Y-maze was employed to test working spatial memory. The Y-maze was composed of two perpendicular arms connected by a start arm. Both of the arms and the starting arm were 45 cm long ×5 cm wide ×15 cm high. The maze was positioned 1 metre above the ground. The two perpendicular arms contained different optical cues (vertical or horizontal stripes). During training the mice were placed in the start arm with access to only one arm for a period of 3 minutes. The training arm alternated between mice. Following a 20 minute intermission in their home-cage, mice were placed back in the Y-maze for 3 minutes with access to both the training and the novel arm. Animals were tracked using Ethovision software (Noldus Information Technology) and the total time spent in the novel arm was analysed.

#### Contextual fear conditioning

The apparatus used for contextual fear conditioning consisted of a chamber (30 cm×20 m×20 cm) with a clear Perspex front wall and ceiling, three stainless steel walls, and a removable shock grid floor consisting of 32 stainless steel rods (0.5 cm diameter, 1 cm apart) with a stainless steel chamber underneath. Chambers were concealed within a sound-attenuating cubicle that contained a 12 V light, an exhaust fan and a video camera. Freezing and locomotor activity were measured using Video freeze software (Med Associates Inc) during both the training and testing sessions. The criterion for a freezing event was set at an observation interval of 2 seconds, where the mouse had to remain under the motion threshold (20 pixels, 30 frames per second) for a period of 500 milliseconds. During training, the mice were placed in the fear conditioning chamber for a period of 20 minutes, during which they received 5 random foot-shocks (0.8 mV). The conditioning context consisted of a 1% acetic acid odour with lights and fans on. Contextual memory was tested 24 hours after training. The mice were placed back in the conditioning chamber for 4 minutes with no shocks under the same contextual conditions as in the initial session.

#### Accelerating Rota-rod

Twenty four hours after the conclusion of contextual fear conditioning, the motor integrity of the mice was tested using the accelerating Rota-rod (UGO Basile Biological Research). Mice were placed on the Rota-rod arm travelling at 2 rpm, accelerating at 0.4 rpm/sec up to a maximum of 20 rpm. Testing was conducted over a 3 minute period and falls, if they occurred before the 3 minute cut-off, were recorded automatically.

#### Sleep/wake cycle

As changes in the integrity of the sleep/wake cycle are a potential confound in the analysis of memory performance, a subset of mice were measured for sleep/wake patterns prior to behavioural testing. Changes in sleep/wake activity were determined by 48 hours of recording via an overhead video camera, and locomotor activity and velocity were measured using Ethovision software. Testing encompassed two full wake cycles and one sleep cycle, with the distance travelled by the mice in 1 hour time bins being used to assess the sleep/wake cycle.

#### Light-dark test

The light–dark test of anxiety was performed using a square open field (30 cm×30 cm×30 cm) made of opaque white acrylic, in which a cardboard insert with a roof was used to enclose half the box. A 5 cm×5 cm hole in the dividing wall allowed access to the enclosed side. Mice were placed in the enclosed side of the box and the number of entries to the open side and the total time spent in the open during a 5 minute test period were recorded.

### Immunohistochemistry

Immediately following behavioural testing mice were deeply anaesthetized with sodium pentobarbital (100 mg/kg i.p.) and transcardially perfused with 20 ml of 0.9% saline containing 1% sodium nitrite and heparin (5000 I.U./ml), followed by 100 ml of 4% paraformaldehyde in 0.1 M phosphate buffer, pH 7.4. Brains were post-fixed overnight and, after repeated washing in phosphate buffered saline (PBS, pH 7.4), placed in 20% sucrose solution for 24 hours. Brains were blocked using a matrix (Stoelting Co.), aligned to the atlas of Franklin and Paxinos [26], and 40 µm coronal sections were cut in three serially adjacent sets through the basal forebrain and hippocampus using a sliding microtome (SM2000r, Leica). Sections were stored in 0.1% sodium azide in 0.1 M PBS.

One series of sections was used to reveal choline acetyl transferase (ChAT) using goat anti-ChAT antibody (1∶3000; Millipore), biotinylated donkey anti-goat IgG (1∶1000; Jackson Immunoresearch Laboratories) and ABC reagent (Vector Elite kit: 6 µl/ml avidin and 6 µl/ml biotin; Vector Laboratories). Black immunoreactive cytoplasm labelled for ChAT was revealed by a nickel-intensified diaminobenzidine reaction, with peroxide being generated by glucose oxidase. Sections were mounted onto chrome-alum/gelatin-treated slides, dehydrated, cleared in histolene, and coverslipped with DePeX (Sigma-Aldrich).

A second series of sections from a subset of mice was used to reveal parvalbumin by immunofluorescence. Free-floating sections were immunostained using mouse anti-parvalbumin antibody (1∶1000; Millipore), and donkey anti-mouse FITC (1∶200; Jackson Immunoresearch Laboratories). Sections were mounted onto chrome alum/gelatin-treated slides and coverslipped with buffered glycerol (pH 8.6).

### Neuronal Counting

Bilateral counts of neurons immunoreactive for ChAT were conducted through the rostro-caudal extent of the basal forebrain. Analysed sections were 120 µm apart. Five sections were analysed for the medial septum, 4 sections for the vertical diagonal band of Broca (beginning 1.42 mm from Bregma), and 5 sections for the horizontal diagonal band of Broca (beginning 0.74 mm from Bregma) and the basal nucleus (Meynerts) (beginning −0.34 mm from Bregma). Four sections of the adjacent nucleus accumbens (beginning 1.42 mm from Bregma) were also analysed [26] All counts were performed by a researcher blind to the experimental conditions.

To determine the specificity of the lesion caused by mu-p75-SAP, counts of parvalbumin-positive neurons through the medial septum and the vertical diagonal band of Broca were performed as above. Eight-bit monochrome images were taken of the basal forebrain sections beginning 1.42 mm from Bregma [26]. Images were captured using a Zeiss Axio Imager Z1 and AxioVision v4.8 software. They were then exported into ImageJ software (NIH) where bilateral counts of parvalbumin-positive nuclei were conducted by a researcher blind to the conditions. Analysis was performed on the mean total counts of ChAT- and parvalbumin-positive cells.

### Analysis

Behavioural and anatomical data were analysed using one-way ANOVA with α set at 0.05 using SPSS software (IBM Corporation). When ANOVA revealed a significant difference between groups, a Tukey post-hoc analysis was performed.

## Results

### p75^NTR^-saporin Induces Specific Lesions of the Basal Forebrain Cholinergic Neurons

**Figure 1:**
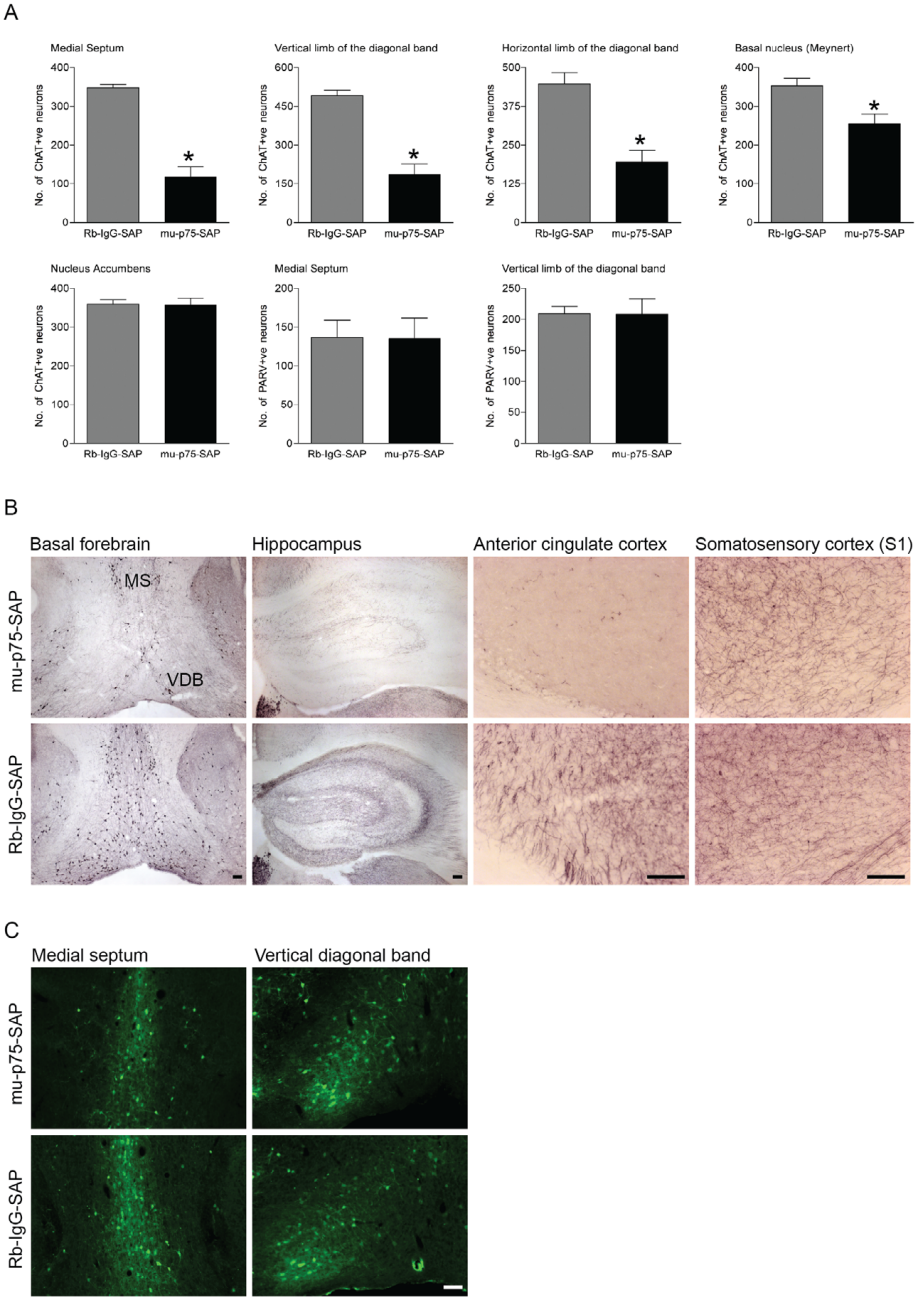
p75^NTR^-saporin induces specific lesions of the basal forebrain cholinergic neurons. (A) Mean (± SEM) numbers of ChAT-positive neurons in basal forebrain nuclei and adjacent nucleus accumbens following intercerebroventricular application of mu-p75-SAP and the control toxin Rb-IgG-SAP. (B,C) Photomicrographs showing (B) ChAT-positive neurons in the medial septum (MS) and vertical limb of the diagonal band of Broca (VDB) and ChAT-positive terminal labelling in the hippocampus, anterior cingulate cortex and primary somatosensory cortex and (C) parvalbumin-positive neurons in the medial septum and vertical limb of the diagonal band of Broca following mu-p75-SAP or Rb-IgG-SAP treatment. (D) Mean (± SEM) numbers of parvalbumin-positive neurons in basal forebrain nuclei following intercerebroventricular application of mu-p75-SAP and the control toxin Rb-IgG-SAP. A specific loss of ChAT-positive neurons was detected in basal forebrain nuclei following mu-p75-SAP treatment (mu-p75-SAP vs. Rb-IgG-SAP, *p<0.001). Scale bars = 100 µm.

To selectively ablate cholinergic basal forebrain neurons we used a saporin-conjugated p75^NTR^ antibody (mu-p75-SAP; [28]–[31]. Intercerebroventricular injection of the murine-specific ribosomal inactivating toxin mu-p75-SAP (0.4 µg) induced a specific loss of cholinergic basal forebrain neurons (Figure 1). As compared to a control injection of Rb-IgG-SAP, the number of ChAT-positive neurons was significantly reduced in the medial septum (F_(1,65)_ = 40.427, p<0.001), vertical diagonal band of Broca (F_(1,65)_ = 38.892, p<0.001), and horizontal diagonal band of Broca (F_(1,65)_ = 19.148, p<0.001). A smaller but significant loss of ChAT-positive neurons was also detected in the basal nucleus (Meynerts) (F_(1,65)_ = 29.88, p<0.001). In contrast, there was no loss of parvalbumin-positive GABAergic neurons in the medial septum or vertical diagonal band of Broca (F_(1,16)_ = 0.065, p = 0.865; F_(1,16)_ = 0.089, p = 0.832, respectively; Figure 1), showing that p75-SAP leads to a selective loss of cholinergic neurons. As expected with the selective loss of ChAT-positive basal forebrain projection neurons, there was an obvious loss of ChAT-positive terminals in both the hippocampus and anterior cingulate cortex but not in the somatosensory cortex (Figure 1 B). There was no change in the number of ChAT-positive neurons in the nucleus accumbens (F_(1,65)_ = 0.004, p = 0.950), demonstrating that the toxin was targeted to p75^NTR^-expressing neurons in the basal forebrain.

### Mice with Cholinergic Basal Forebrain Lesions have Altered Idiothetic Navigation

To assess idiothetic navigation, four groups of mice (mu-p75-SAP+shock, Rb-IgG-SAP +shock, no surgery+shock, and no surgery+no shock) were tested using the place avoidance task. There was no difference in either the distance travelled (F = _(3,21)_ = 0.802, p = 0.507) or speed (F_(3,21)_ = 0.804, p = 0.506) between any of the groups during the 5 minute habituation period, indicating that the lesioned mice did not have any deficits associated with exploring a novel open field. During the training session, conducted 1 hour after habituation, all mice entered a 60° shock zone that was located directly opposite where the animals were introduced to the arena. Under these conditions, all mice that received foot-shocks (0.4 mV) successfully avoided the shock zone for the remainder of the 5 minute training session (data not shown), showing that lesioned animals detected and avoided the shock area during training.

**Figure 2:**
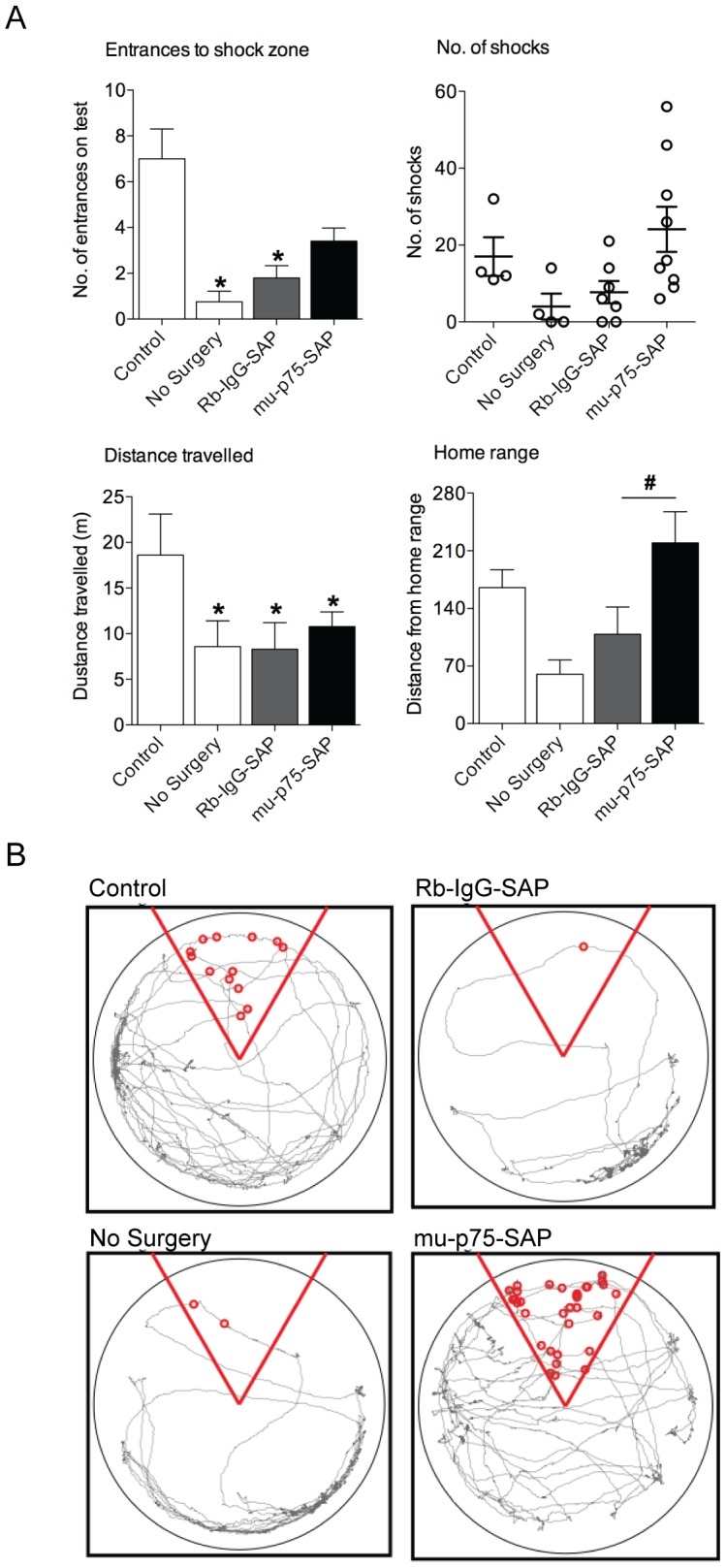
Mice with cholinergic basal forebrain lesions have altered idiothetic navigation. (A) Mean (± SEM) entrances to the shock zone, distance travelled and range of exploratory behaviour from the home zone during the 5 minute passive avoidance test, and scatterplot of the number of potential foot-shock events during this test. Control mice that received a foot-shock during training showed significantly fewer entrances to the shock zone on test than control mice that received no shock during training (* p<0.05). All mice that received a foot-shock during training travelled a significantly shorter distance during test than control mice (* p<0.05). No difference in the distance travelled during test was detected between mice that received a foot-shock during training. Lesioned mice explored further from the home (safe) zone than control mice that received a foot-shock during training (^#^ p<0.05), and showed greater variability in the number of potential foot-shocks during test. (B) Representative tracking of exploratory behaviour during the 5 minute passive avoidance test for each of the four groups.

However, clear differences were apparent between groups at test. Both control groups that received foot-shocks (no surgery+shock and Rb-IgG-SAP+shock) accurately avoided the shock zone during the 5 minute test as compared to the animals that did not receive a foot-shock during training (Figure 2A). Both groups had significantly fewer entrances to the shock zone [Figure 2A; ANOVA (F_(3,21)_ = 8.079, p<0.01), no shock vs no surgery: p<0.05; not shock vs Rb-IgG-SAP: p<0.05]. There was no significant difference in the number of entries on test for lesioned mice as compared to no shock mice (Figure 2A; p = 0.215). However, the behaviour of the lesioned mice on test was clearly different from that of the control mice that did not receive a foot-shock during training, as revealed by the reduced distance travelled on test [Fig. 2A; ANOVA (F_(3,21)_ = 6.389, p<0.01), no shock vs mu-p75-SAP, p<0.05], indicating that their memory for the context was intact. This reduction in exploratory behaviour displayed by the lesioned mice on test was similar to that of control mice that received a foot-shock (mu-p75-SAP vs no surgery: p = 0.805; mu-p75-SAP vs Rb-IgG-SAP: p<0.596; Figure 2A).

Even though lesioned mice travelled the same distance while exploring the arena on test as control mice that received a foot-shock during training, their exploratory behaviour was considerably more random. As the mice were always introduced to the arena directly opposite the shock zone, they learnt that this area of the arena was a safe zone. To quantify the exploratory behaviour of the animals, we measured the range of movement from this home zone. Control animals that received no shocks randomly explored the environment on test (Figure 2A, control). In contrast, animals that had received a foot-shock explored the arena in an organized manner, spending more time in the area closer to the home zone (Figure 2A, no surgery+shock; Rb-Ig-SAP), whereas lesioned animals explored the environment in a more random manner, similar to control animals, and moved much further from the safe zone (Figure 2B; ANOVA F_(3,21)_ = 3.743, p<0.05; mu-p75-SAP vs no surgery+shock: p<0.05; mu-p75-SAP vs Rb-IgG-SAP: p<0.05). Moreover, some lesioned mice randomly entered and stayed within the shock zone as indicated by the large variability in the number of potential foot-shocks received by lesioned mice (Figure 2). This change in exploratory behaviour on test shows that lesioned animals had lost the ability to locate themselves in space.

### Lesioned Animals have Normal Spatial and Contextual Fear Memory

**Figure 3:**
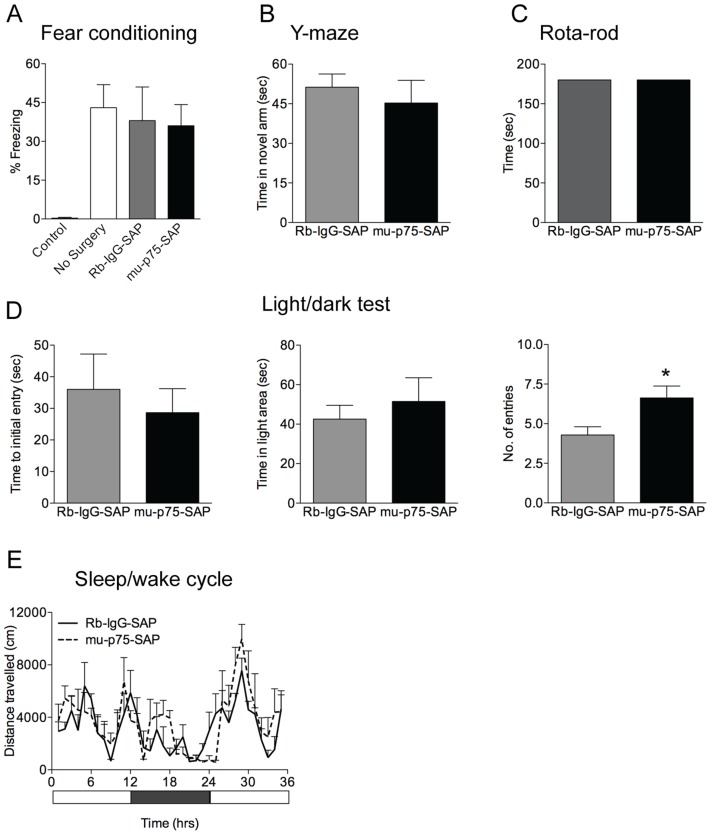
Spatial and contextual fear memory, anxiety, sleep-wake cycles and motor performance of lesioned mice are normal. (A) Mean (± SEM) percent context-elicited freezing 24 hours after fear conditioning training (5 random 0.8 mV foot-shocks). No difference in percent freezing during test was detected in lesioned mice compared to control mice that received foot-shocks,. (B) Mean (± SEM) time spent in the novel arm in the Y-maze. Lesioned mice spent an equivalent amount of time exploring the novel arm of the Y-maze to control mice. (C) Mean (± SEM) time achieved in the accelerating Rota-rod test. All mice successfully completed the 3 minute test with no falls recorded. (D) Mean (± SEM) initial time to enter, total time, and number of entries to the light area in the light/dark test of anxiety. No difference was detected between lesioned and control mice in the initial time taken to enter the light area or in the total time spent in the this area. However, lesioned mice made more entries into the light area during the 5 minute test than control mice (mu-p75-SAP vs Rb-IgG-SAP, *p<0.05). (E) Mean (± SEM) locomotor activity in 1 hour time bins over 36 hours incorporating 2 wake and 1 sleep cycles. No difference in locomotor activity was found between lesioned and control mice in either the wake or sleep cycle.

To determine if the observed behaviour was due to a lack of learning, the spatial working memory of mice was assessed in the novelty seeking Y-maze test. Mice with a basal forebrain lesion spent the same amount of time exploring the novel environment as control mice during the 3 minute test (mu-p75-SAP 45.25 sec ±8.6, Rb-IgG-SAP 51.25 sec ±5.0) (F_(1,7)_ = 0.363, p = 0.569), indicating that in this test of spatial working memory lesioned mice performed as well as control animals (Figure 3B).

Learning was further assessed using contextual fear conditioning. All mice that received the 5 random (0.8 mV) foot-shocks during the training session showed significantly more freezing (F_(3,14)_ = 4.407, p<0.05) and less locomotor activity (F_(3,14)_ = 4.807, p<0.05) than control mice that received no shocks. No differences in the percentage of freezing or locomotor activity were detected in lesioned mice compared to the two control groups that received shocks during training (Freezing: mu-p75-SAP vs Rb-IgG-SAP, F_(3,14)_ = 0.437, p = 0.671 and mu-p75-SAP vs no surgery+shock F_(3,14)_ = 0.951, p = 0.362; Locomotor: mu-p75-SAP vs Rb-IgG-SAP, F_(3,14)_ = 0.065, p = 0.949 and mu-p75-SAP vs no surgery+shock F_(3,14)_ = 0.859, p = 0.409). Similarly, all mice that received foot-shocks during training showed significantly higher levels of freezing and less locomotor activity when they were placed back into the training context (F_(3,14)_ = 7.569, p<0.05 and F_(3,14)_ = 3.518. p<0.05, respectively). Furthermore, no differences in the percentage of freezing or locomotor activity during test were detected in lesioned mice compared to the two control groups that received foot-shocks on training (Freezing: mu-p75-SAP vs Rb-IgG-SAP, F_(3,14)_ = 0.151, p = 0.883 and mu-p75-SAP vs no surgery+shock F_(14,3)_ = 0.529, p = 0.607; Locomotor: mu-p75-SAP vs Rb-IgG-SAP, F_(3,14)_ = 0.177, p = 0.863 and mu-p75-SAP vs no surgery+shock F_(3,14)_ = 0.544, p = 0.597 ) (Figure 3A), indicating that all mice learnt and recalled the contextual information presented to them during training.

### Anxiety, Sleep-wake Cycles and Motor Performance of Lesioned Mice are Normal

To determine whether lesions of the basal forebrain affected levels of anxiety we employed the light/dark test and measured the time taken to enter the light area, the number of entries, and the total time spent in the light. ANOVA showed that the time taken to enter the light, and the total time spent in the light, were comparable between lesioned mice and controls (F_(1,15)_ = 0.311 p = 0.587; F_(1,15)_ = 0.383, p = 0.547, respectively). Interestingly, lesioned mice made more entries into the light side of the apparatus than controls during the 5 minute test (F_(1,15)_ = 6.133, p<0.05) (Figure 3D).

As altered motor performance could be a potential confounding variable, mice that underwent contextual fear conditioning were also assessed for motor performance using the accelerating Rota-rod 24 hours after the conclusion of testing. All mice, regardless of lesion status, reached the 3 minute threshold without any falls from a Rota-rod that accelerated from 2 rpm to 20 rpm at a rate of 0.4 rpm/sec (Figure 3C).

Similarly, a subset of experimental mice were monitored for locomotor activity for 36 hours during two wake and one sleep cycles, as alterations in the sleep/wake cycle can have profound effects on learning and memory performance. No statistical difference in locomotor activity (distance travelled or velocity) was detected by ANOVA (2-way repeated measures with between group factors [mu-p75-SAP vs Rb-IgG-Sap] and repeated measure factor [time]) during the sleep/wake cycle (F_(1,7)_ = 0.005, p = 0.955; F_(1,7)_ = 0.008, p = 0.931, respectively) (Figure 3E).

## Discussion

In this study we used intercerebroventricular application of mu-p75-SAP to induce a discrete and specific loss of basal forebrain cholinergic neurons in the mouse [28]–[31] in order to model the behavioural consequences of degeneration. Although this lesion resulted in a ∼50% loss of medial septum cholinergic neurons that project to the hippocampus, there was no overt histological atrophy of this structure. This lesion therefore closely mimics the cholinergic dysfunction observed in prodromal Alzheimer’s disease [12].

To test the animals’ ability to navigate they were subjected to an idiothetic version of the passive place avoidance test in which all extramaze cues were removed. The design of this task forces the mice to use kinaesthetic self-motion information to locate themselves in space, relative to their home area and the aversive shock zone. Ablation of the basal forebrain cholinergic neurons led to highly disorganized exploratory behaviour. Control mice that received foot-shocks during training successfully avoided the shock zone, and displayed organized exploratory behaviour that centred on the home area of the arena. In contrast, lesioned mice did not stay within the safe area but randomly entered the shock zone, even though they travelled a similar distance to control mice during their exploration.

No deficits in contextual or working spatial memory were apparent following loss of the basal forebrain cholinergic neurons. Lesioned mice showed similar levels of freezing to control mice in contextual fear conditioning, and explored the novel arm of the Y-maze with the same intensity. Furthermore, although lesioned mice were unable to locate the shock zone or home region of the place avoidance arena on test, their behaviour was dramatically different from their performance during habituation, as well as in comparison with that of control mice that were never trained, indicating that their memory for the context was intact. Moreover, we found no deficits in motor performance, anxiety, or the sleep/wake cycle. These findings indicate that the basal forebrain cholinergic system is not critically involved in the cognitive abilities required for context recognition or working spatial memory.

In general, animals use two different strategies to navigate, allothetic and idiothetic. In allothetic navigation the animal uses distal environmental cues to locate itself in space, and continually reorients its motion, whereas in idiothetic navigation the animal uses self-movement cues generated by internal sensory information such as vestibular and proprioceptive input coupled with memory [32]. Previous studies in which the entire medial septal area, encompassing both the cholinergic and GABAergic neuronal populations, was electrolytically ablated in rats reported that these animals had specific deficits in idiothetic navigation [16]. Furthermore, both rats [33] and mice [15] with lesions of the fimbria-fornix, the major pathway carrying cholinergic and GABAergic efferents to the hippocampus, perform poorly in idiothetic navigational tasks. By creating a specific lesion of the basal forebrain cholinergic neurons, our results demonstrate that this system is required for idiothetic navigation.

In a previous study, Moreau et al. [30], using the Morris water maze, reported that, although mice with cholinergic basal forebrain lesions display an early deficit in acquisition in cued navigation, by day three of training they are performing at the same level as control mice and show no deficit during the probe trial. This was interpreted as a spatial memory deficit. However in the Morris water maze, animals have the opportunity to utilize both allothetic and idiothetic navigation strategies. Our results suggest that a more parsimonious interpretation is that mice rely on idiothetic navigation in early trials, whereas allothetic strategies dominate once extramaze cues become learnt. Furthermore, Moreau et al. [30] reported that lesioned mice had an increased incidence of thygmotaxic search strategies and were slower at adopting a goal-directed strategy, consistent with the animals exhibiting impaired idiothetic navigation.

A major function of basal forebrain hippocampal innervation is the generation of the 4–10 Hz theta rhythm. Lesion studies targeting GABAergic or cholinergic inputs to the hippocampus have revealed that the GABAergic system is responsible for the pacing of theta rhythm synchronization, whereas the cholinergic system contributes to the amplitude or the power of the rhythm [34]. Although the hippocampal theta rhythm is active during voluntary movement and has been implicated in attention and acquisition of sensory information [35], [36], only recently has vestibular stimulation, sensory information critical for idiothetic navigation, been shown to stimulate this rhythm [37]. Moreover, the locomotion-induced increase in theta amplitude can be blocked by the muscarinic acetylcholine receptor antagonist atropine, consistent with a role for the basal forebrain cholinergic neurons in transmitting this sensorimotor information to the hippocampus.

It is well established, based on single-cell recordings, that hippocampal neurons respond to movement, place, and head orientation [38], [39]. Therefore, the hippocampus plays a significant role in the generation of spatial awareness and the coding of self-movement through efferent copies of movement-related commands. In light of our evidence that basal forebrain cholinergic neuron lesions produce a specific deficit in idiothetic navigation, it is likely that the amplitude of the theta rhythm modulated by acetylcholine [34], [40] plays a critical role in the processes required to be aware of where you are in space relative to where you have been. Failure of appropriate hippocampal integration of this self-movement information due to atrophy of the basal forebrain cholinergic neurons or dysregulation of acetylcholine receptor function in the hippocampus, as occurs in Alzheimer’s disease [41], [42], could underpin deficits in this form of navigation.

Both basal forebrain atrophy and hippocampal volume loss, as measured by magnetic resonance imaging, are emerging as accurate markers for diagnosing at risk Alzheimer’s disease patients, with basal forebrain atrophy and hippocampal volume loss being highly correlated [11], [14]. Furthermore, poor navigation in patients with MCI is correlated with hippocampal atrophy [7]. The contribution of the basal forebrain cholinergic system to these spatial memory deficits is yet to be established in humans. However, our finding that an idiothetic navigational deficit is caused by a loss of basal forebrain cholinergic neurons in mice provides strong evidence-based support for the examination of basal forebrain cholinergic neuron dysfunction, measured with either magnetic resonance imaging or behavioural navigation tasks, as an early diagnostic marker for the cognitive decline that is associated with Alzheimer’s disease.
